# Development of a machine learning model to estimate length of stay in coronary artery bypass grafting

**DOI:** 10.11606/s1518-8787.2024058006161

**Published:** 2024-09-16

**Authors:** Renato Camargos Couto, Tania Pedrosa, Luciana Moreira Seara, Vitor Seara Couto, Carolina Seara Couto

**Affiliations:** 1 Faculdade de Ciências Médicas de Minas Gerais. Fundação Lucas Machado. Belo Horizonte, MG, Brasil; 2 Instituto de Acreditação e Gestão em Saúde. Departamento de Ciências de Dados. Belo Horizonte, MG, Brasil; 3 Hospital Unimed. Belo Horizonte, MG, Brasil; 4 Hospital Governador Israel Pinheiro. Belo Horizonte, MG, Brasil; 5 Instituto de Previdência dos Servidores do Estado de Minas Gerais. Belo Horizonte, MG, Brasil

**Keywords:** Length of Stay, Machine Learning, Coronary Artery Bypass

## Abstract

**OBJECTIVE:**

To develop and validate a predictive model utilizing machine-learning techniques for estimating the length of hospital stay among patients who underwent coronary artery bypass grafting.

**METHODS:**

Three machine learning models (random forest, extreme gradient boosting and neural networks) and three traditional regression models (Poisson regression, linear regression, negative binomial regression) were trained in a dataset of 9,584 patients who underwent coronary artery bypass grafting between January 2017 and December 2021. The data were collected from hospital discharges from 133 centers in Brazil. Algorithms were ranked by calculating the root mean squared logarithmic error (RMSLE). The top performing algorithm was validated in a never-before-seen database of 2,627 patients. We also developed a model with the top ten variables to improve usability.

**RESULTS:**

The random forest technique produced the model with the lowest error. The RMLSE was 0.412 (95%CI 0.405–0.419) on the training dataset and 0.454 (95%CI 0.441–0.468) on the validation dataset. Non-elective surgery, admission to a public hospital, heart failure, and age had the greatest impact on length of hospital stay.

**CONCLUSIONS:**

The predictive model can be used to generate length of hospital stay indices that could be used as markers of efficiency and identify patients with the potential for prolonged hospitalization, helping the institution in managing beds, scheduling surgeries, and allocating resources.

## INTRODUCTION

Coronary artery bypass grafting (CABG) is the most commonly performed cardiac surgery in Brazil, representing 54.1% of cases^1^. The length of hospital stay (LOS) related to CABG serves as a significant indicator of the quality of institutions providing this service, as it directly impacts resource utilization and healthcare costs. Timely discharge of patients following CABG can facilitate hospital workflow by freeing up beds and optimizing healthcare professionals’ time^2^. Furthermore, an unnecessarily prolonged hospital stay can escalate the likelihood of adverse events such as falls, hospital infections and medication errors. The development of a predictive model capable of estimating LOS can be an effective tool for managing hospital expenses, enhancing service efficiency, and ensuring better patient care^3^.

Traditionally, predictive models in medicine are made by algorithms based on traditional statistics^4^. However, in recent times, machine learning algorithms have emerged as a viable approach for developing predictive models^5-7^. Determining the most effective model for generating accurate predictions involves comparing different algorithms’ predictive abilities.

A potential limitation of predictive models is that they may not be applicable to a patient population that significantly differs from the population in which the model was developed^8^. To the best of our knowledge, no predictive models specifically developed to estimate LOS for patients undergoing CABG have been trained and validated using a Brazilian database.

The primary objective of this study was to develop and validate a predictive model utilizing machine learning techniques for estimating LOS among patients who underwent CABG, utilizing data from a Brazilian administrative database.

## METHODS

### Source of Data and Study Participants

For training and validation, we used an administrative database collected from hospital discharges from 133 hospitals, in all regions of Brazil, from January 2017 to December 2022. The database is derived from public and private insurance hospitals that employ DRG for the management of healthcare processes in healthcare systems. The dataset contains information for each hospitalization, encompassing demographic details, adverse events, primary and secondary diagnosis codes, as well as procedure codes. Data were collected by healthcare professionals trained in data collection. The entire database of patients who underwent CABG, comprising 12,211 admissions of individuals aged 18 years and older, was utilized to enhance the power of the predictive model and its generalization. Data from 9,584 patients who underwent CABG with or without valve replacement between January 2017 and December 2021 were used for training and internal validation of the predictive model. Then, the predictive model with the best performance was validated in a new sample of 2,627 patients who had undergone CABG between January 2022 and December 2022. Exclusion criteria were patients younger than 18 years, patients transferred to another hospital and patients who died in the same hospitalization. The study followed the Transparent Reporting of the Multivariable Prediction Model for Individual Prognosis or Diagnosis (TRIPOD) guidelines^9^.

### Outcome and Variable Selection

The variables were collected at hospital discharge using the International Classification of Diseases, 10^th^ Revision (ICD-10), coding. Coders were instructed to identify conditions present on admission. Because there is a great number of ICD-10 codes, we categorize them into clinically meaningful groups. In total, 59 independent variables were included in the predictive model. We also developed a model with the top ten variables identified by Shapley Additive Explanations (SHAP) technique^10^. Variables that the authors considered not to be clinically plausible predictors of LOS stay were excluded. The outcome was LOS for patients undergoing CABG. LOS in days was calculated from the difference between the date and time of admission and discharge.

### Missing Data

Data from 88 patients were missing in the database. Patients with missing data were excluded from the analysis. We considered that the missing data occurred completely at random and that they represented a small part of the sample.

### Model Training, Selection, and Validation

The training sample was used to train three machine learning models (random forest, extreme gradient boosting and neural networks) and three traditional regression models (Poisson regression, linear regression, negative binomial regression). To guide algorithm selection, we ranked the predictive capacities of models. The model with the best performance was selected and validated in a new sample of patients undergoing CABG at a distinct time. For training the predictive models, a sample of 9,584 patients undergoing CABG between January 2017 and December 2021 was used. For machine learning models, 30% of the training base was selected for a hold out for internal validation and excluded from the database. In the remaining portion of the database, we used the K-fold cross validation method^11^: the dataset was divided into ten folds, with each algorithm being trained ten times. In each iteration, one fold was utilized for validation while the remaining nine folds were used as the training dataset. The process continues until all parts have participated in both the training and validation processes. This procedure has a single parameter k, which refers to the number of groups the training dataset should be divided into for training and validation purposes. The most common values used for k range from 5 to 10. We used k = 10 in all tested models.

The model with the best performance was defined through the root mean squared and logarithmic error (RMLSE) in the training dataset. The RMLSE was chosen as our metric because it penalizes the model more severely when the predicted value is less than the actual value compared to when the predicted value is more than the actual value, but we also included other common regression metrics of the models in a supplemental materiala. The closer the RMLSE is to 0, the smaller the error of the model^12^. While it may not be the most optimal method for evaluating the predictive accuracy of machine learning models, we calculated the R² value for the top-performing model to facilitate a comparative analysis against alternative predictive models. Internal validation was performed on 30% of the training base that had been separated for this purpose. We also developed a model with the top ten variables identified by SHAP technique in the validation dataset, to improve usability.

The algorithm that exhibited the highest performance using all variables and the model employing the top ten variables were both trained on the entire training dataset. The models were then validated in a never-before-seen database of 2,627 patients who had undergone CABG between January 2022 and December 2022.

To set the hyperparameters of the chosen model, we used the Grid Search technique. A set of possible values for each hyperparameter was selected and all possible combinations of these hyperparameters were tested. The hyperparameters that obtained the best performance were used for the final model. Tuning hyperparameters prevents the model from learning solely from the presented data (avoiding overfitting and underfitting), enabling it to generalize to other possible scenarios^13^.

The SHAP technique was used to define the relative importance of each model variable in the outcome. This is done by comparing the predictive ability of a model with and without a given variable. The larger the difference between the predictions, the more important the variable is to the model. The advantage of using SHAP values lies in the fact that they add interpretability to complex models^10^.

A calibration curve was also built to assess the predictive ability of the selected model. The main objective of a calibration curve is to assess the alignment of model predictions with the true values of the output variable. It provides an idea of how well the model's predictions are aligned with the actual values when comparing numerical predictions with the true values of the outcome variable. To do this, predictions are grouped into intervals or bins, creating groups of similar predictions. An ideal calibration curve would have all points located on the diagonal line, indicating perfect calibration of predictions with respect to actual values. By observing the calibration curve, it is possible to identify whether the model is consistently underestimating or overestimating the values, which may indicate the need for adjustments or refinements in the model^14^.

To describe the characteristics of patients, we used the Mann-Whitney test to compare continuous variables and Pearson's χ² test to compare categorical variables. For each of the most important variables, we compared the LOS between patients with and without that characteristic. Since the LOS did not follow a normal distribution, we employed a non-parametric test (Mann-Whitney) to assess whether the difference in hospital stay was significant between patients with the characteristic and those without it, using a significance level of 5%.

This study was approved by the Ethics and Research Committee of the Medical Sciences School of Minas Gerais, Brazil (Certificate of Presentation for Ethical Appreciation — CAEE: 29000819·0.0000·5134). It was classified as a low-risk study, since it used anonymous convenience samples extracted from a database which is used for managerial purposes. The study did not require participants to sign the informed consent form. Python software (version 3.7) was used for training and validation of the model.

## RESULTS

### Patient Characteristics

**Figure 1:**
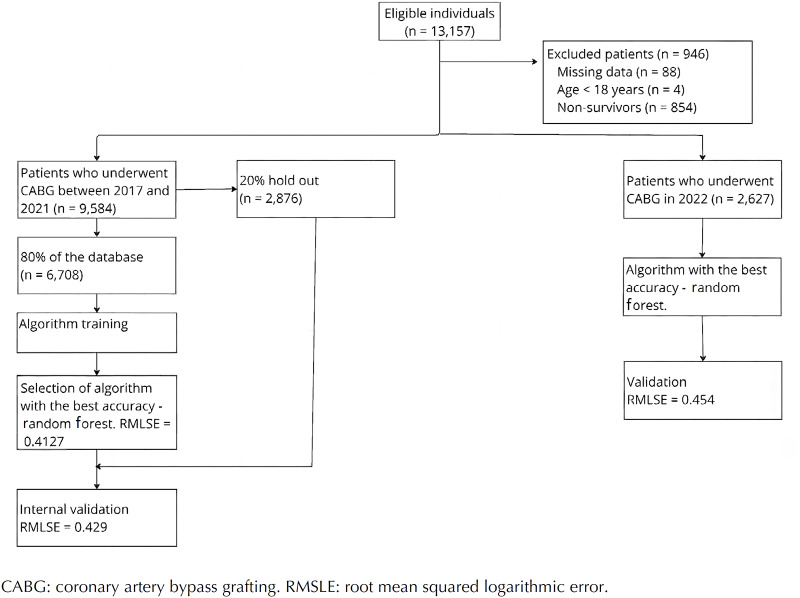
Flow of participants throughout the study.

Data from 9,584 patients (75% male, mean age 63.2 (9.6) years) who underwent CABG between January 2017 and December 2021 were utilized for training the algorithm. Subsequently, the algorithm's performance was assessed using a new dataset comprising 2,627 patients (74.2% male, mean age 63.7 (9.4) years) who underwent CABG between January 2022 and December 2022. The flow of participants through the study is depicted in Figure 1. Baseline characteristics of both the training and validation cohorts, along with their unadjusted association with the primary outcome, are presented in Table 1. The characteristics of the training and validation groups were similar. The average duration of hospital stay was 14.8 days (standard deviation - SD = 10.3) for the training cohort and 15.9 days (SD = 12.6) for the validation sample. Out of the total surgeries considered in this study, 55.9% (6,826 patients — 5,363 on the training and 1,463 on the validation dataset) were classified as non-elective procedures. Valve replacement was performed in 11.8% (1,436 patients — 1,136 on the training and 300 on the validation dataset) of the surgeries, and 52.0% (6,345 patients — 4,398 on the training and 1,407 on the validation dataset) of the hospitalizations occurred within the Brazilian public health system (SUS). The average number of chronic health conditions per patient was 4.26 (SD = 1.97). Among the patients included in this study, 2,654 patients (21.7%) had more than five chronic health conditions. The in-hospital mortality rate for patients who underwent CABG was 6.5% (854 patients).

CABG: coronary artery bypass grafting. RMSLE: root mean squared logarithmic error.

**Table 1:** Characteristics of patients used for algorithm training and validation.

Variable			Training database				Validation database			
			Total	LOS(SD)	MedianLOS	p-value^a^	Total	LOS(SD)	Median LOS	p-value^a^
Total admissions			9,584	14.8 (10.3)	12	-	2,627	15.9 (12.6)	12	
Sex, n (%)						< 0.001				< 0.001
		Female	2,393 (25.0)	16.7 (12.3)	13		678 (25.8)	17.3 (11.6)	13	
		Male	7,191 (75.0)	14.2 (9.5)	12		1,949 (74.2)	15.5 (14.1)	12	
Age at surgery, mean (SD), years			63.2 (9.6)	-	-	-	63.7 (9.4)	-	-	
Elective surgery, n (%)						< 0.001				< 0.001
		No	5,363 (56.0)	18.3 (11.1)	16		1,463 (55.7)	20.7 (13.7)	18	
		Yes	4,221 (44.0)	10.4 (7.2)	8		1,164 (44.3)	9.9 (7.5)	8	
Payer source, n (%)						< 0.001				< 0.001
		Public health	4,938 (51.5)	16.9 (11.0)	15		1,407 (53.6)	19.3 (13.9)	16	
		Private health	4,559 (47.6)	12.7 (9.0)	10		1,220 (45.8)	12.1 (9.5)	9	
Valve replacement						< 0.001				< 0.001
		Yes	1,136 (11.9)	17.5 (14.1)	13		300 (11.4)	18.7 (15.5)	14	
		No	8,448 (88.1)	14.5 (9.7)	12		2,327 (88.6)	15.6 (12.1)	12	
Comorbidities										
		Heart failure, n (%)				< 0.001				< 0.001
		Yes	1,199 (12.5)	19.1 (12.8)	16		388 (11.4)	20.5 (14.8)	17	
		No	8,385 (87.5)	14.2 (9.7)	12		2,239 (85.2)	15.1 (11.9)	12	
		Non-STE ACS, n (%)				< 0.001				< 0.001
		Yes	4,628 (48.3)	16.1 (10.4)	14		1,249 (47.5)	17.6 (12.4)	15	
		No	4,956 (51.7)	13.6 (10.1)	10		1,378 (52.5)	14.4 (12.5)	10	
		STE ACS, n (%)				< 0.001				
		Yes	707 (7.4)	20.5 (10.2)	19		212 (8.1)	25.3 (14.3)	22	
		No	8,877 (92.6)	14.4 (10.2)	12		2,415 (91.9)	15.1 (12.1)	11	
		Chronic kidney injury, n (%)				< 0.001				< 0.001
		Yes	435 (4.5)	21.2 (16.4)	17		127 (4.8)	23.4 (16.9)	18	
		No	9,149 (95.5)	14.5 (9.9)	12		2,500 (95.2)	15.4 (12.2)	12	
		AF/Flutter, n (%)				< 0.001				< 0.001
		Yes	843 (8.8)	18.5 (13.7)	15		255 (9.7)	20.7 (16.0)	16	
		No	8,741 (91.2)	14.5 (9.9)	12		2,372 (90.3)	15.4 (12.0)	12	
		Acute respiratory failure, n (%)				< 0.001				< 0.001
		Yes	300 (3.1)	22.3 (14.9)	18		112 (4.3)	29.4 (22.2)	23	
		No	9,284 (96.9)	14.6 (10.1)	12		2,515 (95.7)	15.3 (11.6)	12	
		Acute kidney injury, n (%)				< 0.001				< 0.001
		Yes	191 (2.0)	25.5 (15.1)	22		92 (3.5)	26.7 (17.7)	22	
		No	9,393 (98.0)	14.6 (10.1)	12		2,535 (96.5)	15.5 (12.2)	12	
		ID DM, n (%)				< 0.001				< 0.001
		Yes	844 (8.8)	17.4 (12.8)	14		229 (8.7)	20.7 (17.2)	16	
		No	8,740 (91.2)	14.6 (10.1)	12		2,398 (91.3)	15.5 (11.9)	12	
		COPD				<0.001				< 0.001
		Yes	370 (3.9)	19.0 (13.3)	16		112 (4.3)	24.2 (19.9)	18.5	
		No	9,214 (96.1)	14.6 (10.2)	12		2,515 (95.7)	15.6 (12.0)	12	
		Mitral valve disease, n (%)				< 0.001				< 0.001
		Yes	504 (5.3)	19.6 (15.2)	16		160 (6.1)	20.4 (15.4)	16.5	
		No	9,080 (94.7)	14.5 (9.9)	12		2,467 (93.9)	15.6 (12.3)	12	
		Dyslipidemia, n (%)				< 0.001				0.003827
		Yes	3349 (34.9)	14.2 (9.5)	12		807 (30.7)	15.0 (11.9)	11	
		No	6,235 (65.1)	15.2 (10.9)	13		1,820 (69.3)	16.3 (12.9)	13	
		Acute pulmonary edema, n (%)				< 0.001				< 0.001
		Yes	223 (2.3)	21.7 (17.3)	18		59 (2.2)	22.4 (13.7)	21	
		No	9,361 (97.7)	14.6 (10.1)	12		2,568 (97.8)	15.7 (12.5)	12	
		Other arrhythmias, n (%)				< 0.001				0.01154
		Yes	252 (97.4)	18.5 (15.4)	15		61 (2.3)	20.5 (17.7)	16	
		No	9332 (2.6)	14.7 (10.2)	12		2,566 (97.7)	15.8 (12.4)	12	
		COVID-19, n (%)				< 0.001				< 0.001
		Yes	72 (0.8)	28.6 (17.5)	24		26 (1.0)	30.2 (12.5)	31.5	
		No	9,512 (99.2)	14.7 (10.2)	12		2,601 (99.0)	15.8 (12.5)	12	
		NID DM, n (%)				0.2925				0.1621
		Yes	3,028 (31.6)	14.7 (10.4)	12		819 (31.2)	15.7 (13.5)	12	
		No	6,556 (68.4)	14.8 (10.3)	12		1,808 (68.8)	16.0 (12.1)	13	
		Hypertension, n (%)				0.3058				0.1462
		Yes	7,510 (78.4)	14.7 (10.1)	12		2,014 (76.7)	16.0 (12.5)	12	
		No	2,074 (21.6)	15.2 (11.2)	12		613 (23.3)	15.5 (12.9)	12	
		Obesity, n (%)				< 0.001				0.001246
		Yes	893 (9.3)	16.3 (11.3)	13		236 (9.0)	18.6 (16.3)	15	
		No	8,691 (90.7)	14.5 (10.2)	12		2,391 (91.0)	15.7 (12.1)	12	
		Drug use, n (%)				< 0.001				0.02492
		Yes	198 (2.1)	22.3 (9.7)	21		21 (99.2)	17.9 (6.9)	19	
		No	9,386 (97.9)	14.7 (10.3)	12		2,606 (0.8)	15.9 (12.6)	12	

LOS: length of hospital stay; SD: standard deviation; Non-STE ACS: non-ST elevation acute coronary syndrome; STE ACS: ST elevation acute coronary syndrome; AF: atrial fibrillation; ID DM: insulin dependent diabetes mellitus; NID DM: non-insulin dependent diabetes mellitus; COPD: chronic obstructive pulmonary disease).

a Mann-Whitney test for significant differences between groups.

### Model Performance

We trained three machine learning models (random forest, extreme gradient boosting and neural networks) and three traditional regression models (Poisson regression, linear regression, negative binomial regression) in the training dataset. The random forest model had the best performance and was then validated in a new dataset, followed by the extreme gradient boosting model.

**Figure 2:**
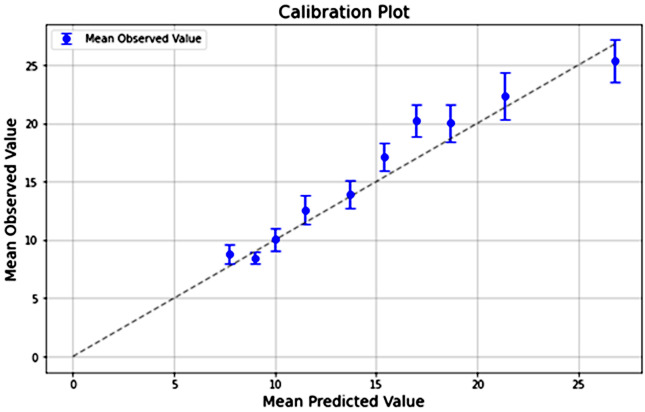
Calibration plot of validation dataset. Calibration curve demonstrating the predicted lenght of stay relative to actual lenght of stay for the model.

The RMLSE of the random forest model was 0.412 (95%CI 0.405-0.419) on the training dataset, 0.429 (95%CI 0.417-0.440) on the hold-out and 0.454 (95%CI 0.441-0.468) on the validation on a distinct dataset collected between January 2022 and December 2022. The RMLSE of the top ten model was 0.485 (95%CI 0.470-0.500) on the validation on a distinct data set and was not significantly different from the model which included all the variables (Signed Wicoxon Test, p-value = 1). Model calibration curves on the validation dataset are shown in Figure 2. The model with the top ten variables is available for use through the link: https://rvsm.reiks.tec.br/

### Feature Importance

**Figure 3:**
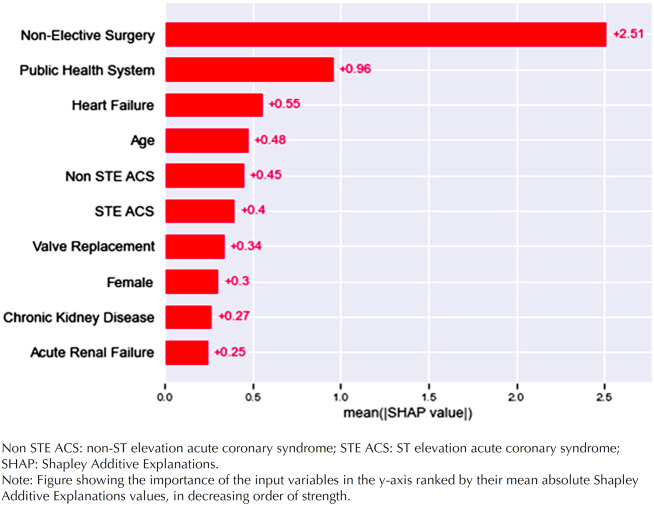
Shapley Additive Explanations analysis in validation dataset.

The SHAP analysis showed that the variables with the greatest impact on the patient's LOS in decreasing order of strength were non-elective surgery, admission to a public hospital, presence of heart failure, age, and Non-STE-ACS. The strongest variables for model prediction were similar in the training and validation datasets (Figure 3).

Non STE ACS: non-ST elevation acute coronary syndrome; STE ACS: ST elevation acute coronary syndrome; SHAP: Shapley Additive Explanations. Note: Figure showing the importance of the input variables in the y-axis ranked by their mean absolute Shapley Additive Explanations values, in decreasing order of strength.

**Figure 4:**
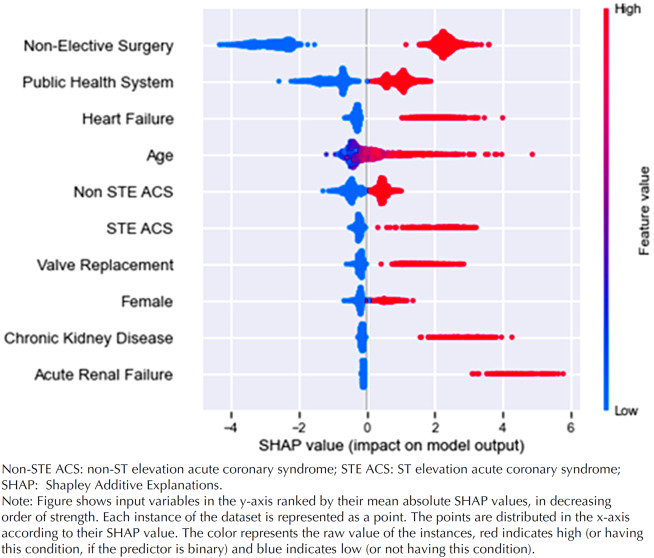
Summary plot showing variables’ relative importance and their effect on the predicted outcomes within the model in the validation dataset.

A summary plot showing the variables’ relative importance and their effect on the predicted outcomes within the model in the validation dataset is shown (Figure 4).

## DISCUSSION

The machine-learning system may experience diminished performance when there is a mismatch between the dataset with which it was developed and the data on which it is deployed, in a process called dataset shift^15-17^. A machine learning model trained on a specific population or healthcare structure can underperform when applied in a new setting^8^. To the best of our knowledge, this is the first predictive model developed to estimate LOS in patients undergoing CABG using a Brazilian database.

Our study used an administrative database to produce an machine learning model capable of estimating LOS in patients undergoing CABG with RMLSE = 0.454 for the validation of the model including all variables and RMLSE = 0.485 for the validation of the top ten model. The model can reliably predict the LOS in groups of patients, as demonstrated in the calibration curve. The algorithm was developed using data collected from multiple centers across all regions of Brazil, encompassing a large population. Through direct comparison of different predictive models of machine learning and traditional statistics, it was possible to avoid bias associated with algorithm selection. The random forest technique produced the model with the lowest error. Other strengths of our study include a large sample size and the utilization of LOS as a continuous variable rather than a categorical one. A model with the top ten variables was not significantly different from the model which included all the variables, improving usability. Our algorithm with the top ten variables is freely accessible via a provided weblink, facilitating the dissemination of the algorithm to centers that can utilize a model adjusted to local realities and further validate it.

Traditional statistics models may incorrectly presuppose linear interactions among the variables influencing outcomes, thereby constraining the effectiveness of predictive models^18^. In contrast, machine learning approaches consider the dynamic interplay of variables in ways that are nonlinear and nonparametric, leading to enhanced prediction tools^19^. Machine learning employs more flexible techniques that facilitate the incorporation of extensive volumes of multidimensional data^20^. Supporting this assertion, the random forest technique yielded a model with better performance compared to the conventional statistical models (Poisson regression, linear regression, negative binomial regression) we developed in our database.

The random forest algorithm is a powerful Machine learning technique that can be used for both regression and classification models. It is a special case of an ensemble method called «bagging» (bootstrap aggregating). Bagging involves training multiple models on different random subsets of the original dataset and combining their predictions to produce a more robust result. In the case of random forest, bagging is applied to a set of decision trees. Multiple training subsets are created from the original dataset using a technique called «bootstrap sampling» for each subset. For each training subset, a decision tree is trained. The predictions from all individual trees are combined to form a final prediction^20^. In the case of random forest, the combination often involves averaging (for regression) or voting (for classification) the predictions of individual trees. The key feature of random forest is the introduction of randomness during tree construction. This includes the random selection of features at each node split and the use of random feature subsets for each tree. The introduced randomness helps reduce the correlation between individual trees, making the ensemble more diverse and, therefore, less prone to overfitting. This diversity is fundamental for improving the generalization of the model^19^.

Most of the studies examining LOS in patients who undergo CABG solely focused on the duration of their stay in the intensive care unit^21-24^. Alshakhs et al.^25^ conducted a study with a predictive model using Machine learning for hospital LOS after CABG in Saudi Arabia with 621 patients. In contrast to our study, LOS was categorized into high and low risk. The random forest technique also produced the model with the best accuracy. Osnabrugge et al.^26^ developed a predictive model for hospital LOS and costs in patients undergoing CABG using linear regression analysis in a large database. Triana et al.^27^ developed a Machine learning model for LOS using data from a single institution's Society of Thoracic Surgeons (STS) Registry. Our model had a better R² coefficient (R² = 0.277 for the validation of the model including all variables, R² = 0.1933 for the top ten model) than these other models that considered only preoperative variables (R² = 0.10 and R² = 0.058, respectively). Both studies also developed a model including preoperative, intraoperative, and postoperative variables, with R² values of 0.51 and 0.232, respectively. Recognizing that predictions should be adjusted for variations in risk factors as opposed to differences in outcomes, we made a choice to exclude these variables. This decision aligns with a key focal point of our model, which is benchmarking to be used in management improvement programs and the establishment of pay-for-performance mechanisms. It is important to highlight that the utilization of R² might not be the most suitable approach for assessing the predictive precision of intricate models such as random forests. R² quantifies the extent to which independent variables in the model account for the variability in the dependent variable. However, machine learning methods can grasp non-linear and interactive elements—features that conventional linear regression models might miss. This distinction leads to R^2^'s limitations when it comes to capturing the nuances of complex relationships that random forests can handle^28^.

The risk factors for prolonged LOS that we found are similar to previous studies. Osnabrugge et al.^26^ and Almashrafi et al.^29^ also described non-elective surgery as a risk factor for increased LOS. Patients with heart failure also had longer LOS in the studies conducted by Almashrafi et al.^29^ and Lazar et al.^30^ Combined CABG and valve surgery was also found to increase LOS in the study conducted by Almashrafi et al.^29^ Triana et al.^27^ also found age to increase LOS. One of the main risk factors for prolonged LOS found in our study was performing CABG in the public health system, which may reflect the influence of the low income of the population assisted in this system, or its poorer quality of structure and hospital processes. We did not find another study that analyzed this variable.

Our study has several limitations. Despite using a database collected in a different period to validate the model, this was a retrospective database collected from the same institutions where the model was trained. Furthermore, the characteristics of the patients in the training and validation samples are very similar, probably leading to overly optimistic results. It would be important to carry out an external and prospective validation, ideally assessing the application of the model by clinicians in clinical practice. Access to other variables could increase the explanatory power of our model. As this is a database collected for administrative purposes, we did not have access to risk factors that influenced LOS in other studies, such as the use of intra-aortic balloon pump, laboratory results, use of intravenous nitroglycerin and pulmonary artery systolic pressure^22,24,25,27^. Building a specific database for conducting studies in patients undergoing cardiac surgery could further enhance the predictive capacity of the model. Other studies have indicated that postoperative factors and adverse events are significant contributors to increased length of stay (LOS)^26,27^. However, incorporating such variables would hinder the model suitability for hospital benchmarking and pay-for-performance measures. We excluded non-survivors to predict LOS. Since the characteristics of patients who survive are different from those of patients who die in hospital, our findings could be altered in a population including non-survivors. This affects the generalizability of our algorithm.

Hospital LOS is an important marker of quality of care. The developed model can be used to compare the observed and expected LOS to generate LOS indices that could be used as markers of efficiency, allowing healthcare systems to manage resources and optimize hospital payment models. In addition, identifying patients with the potential for prolonged hospitalization can help the institution in managing beds, scheduling surgeries, and allocating resources. The predictive model can also help in the psychological preparation and planning of the patient and his family.
