# Supplementary material for: Development of a machine learning model to estimate length of stay in coronary artery bypass grafting
Source: Rev Saude Publica. 2024 Sep 16;58:41. doi: 10.11606/s1518-8787.2024058006161 (PMC11578580; doi:10.11606/s1518-8787.2024058006161)
Supplement: Supplementary file 1 [file 1518-8787-rsp-58-41-Suppl01.docx]

|  | Randon Forest | XGBoosting | Neural Network | Poisson Regression | Negative Binomial Regression | Linear Regression |
| --- | --- | --- | --- | --- | --- | --- |
| RMSLE | 0.412 | 0.429 | 0.435 | 0.440 | 0.443 | 0.433 |
| MAE | 5.175 | 5.303 | 5.461 | 5.526 | 5.598 | 5.413 |
| MSE | 68.235 | 67.581 | 78.277 | 76.335 | 78.205 | 83.621 |
| RMSE | 8.260 | 8.221 | 8.847 | 8.737 | 8.843 | 9.144 |

**Table 1.** Regression metrics used in the 6 evaluated models in training dataset (RMSLE = Root Mean Squared Logarithmic Error; MAE = Mean Absolute Error; MSE = Mean Squared Error; MAE = Mean Absolute Error; RMSE = Root Mean Squared Error)
